# How to do a person-centred eye health consultation

**Published:** 2015

**Authors:** Renée du Toit

**Affiliations:** Consultant, Pretoria, South Africa. Email: dutoitrenee@gmail.com

**Figure:**
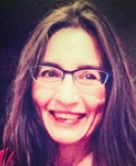
Renée du Toit

The care we give should focus on our patients – their needs, beliefs, and preferences – and not just on their disease. This is known as **patient-centred** care. We can take this idea further and talk about **person-centred** care, which reminds us that we should be concerned with the whole person – and their life – when they are outside of the clinic too, not just when they are in front of us.

## Improving access

Before person-centred eye care can start, eye health services must be made accessible to all. This can be done by finding out about community needs, encouraging people to come for eye services, and making the service child friendly and accessible to older people and those with low vision, other disabilities, and mobility impairments. Previous issues of the *Community Eye Health Journal* have discussed all of the above in detail.

## Engaging people in their own health care

Person-centred care does not mean that people are left to independently make medical decisions. What it does mean, is that people are active partners in these decisions.

We are all different, and person-centred care enables people to actively contribute to their own personalised care, treatment or support over time, both during the consultation and afterwards, in a way that is appropriate to their needs and circumstances. Having a person-centred focus can improve care and result in better outcomes. For example, people with chronic eye diseases such as glaucoma must be involved in their eye health in a very active way, by returning to the clinic for regular follow-up examinations and either coming for laser or surgery, or instilling eyedrops every day.

Here are some tips for providing a patient-centred eye consultation.

1. **Consider the patient as a whole** From when you first see the person, note their physical and behavioural characteristics. Are there signs that indicate the person has eye pain? Observe how the person uses their vision when they come into the room. Take these into account throughout the consultation and assist people with visual or other disabilities as required.
  **Figure:**
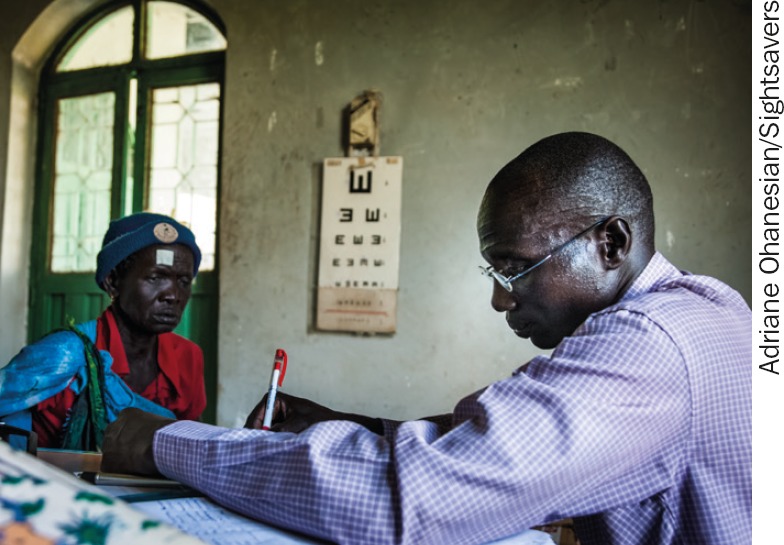
A person-centred focus can improve care. SOUTH SUDAN
2. **Establish a good relationship and use respectful communication to promote trust** Greet the person well and have a respectful, empathetic and compassionate manner. Get to know the person as an individual. Take time to listen. Be considerate of people with impairments, whether hearing, visual, or intellectual (e.g. a learning disability).
3. **Take a history – find out what the person understands is their problem and what they expect** Ask the person to tell you the reason for the visit. This will guide the history taking and help you to get the information you need.
  Find out what the person's concerns are. These could include pain and/or ability to function. The concerns could also be non-medical, such as cost, time spent waiting, oran inability to come for a repeat visit.
  Ask about the person's expectations of treatment and outcome; this will help you to understand her or his point of view and needs.
4. **Do an eye examination and assess vision** Undertake a full eye examination, as appropriate for the history.
  Based on this information, explain to the person what further tests are necessary, how long these will take, and what these will cost. If a referral to another health centre or hospital is needed, ensure that they have all the information they need to be able to successfully take up the referral.
5. **Decide on a treatment plan in discussion with the patient/family** Discuss tests, diagnosis, treatment and prognosis in an open and non-judgemental way to ensure that the patient and her/his family understand what they have been told about the condition.
  - Ask the person if there is anything they do not understand.
    **Before performing any eye procedure**
    - Wash your hands (and afterwards too).
    - Use gloves if indicated e.g. for invasive procedures or if an eye appears infectious.
    - Wipe / disinfect equipment.
    - Ensure that lighting is appropriate
    - Clearly explain to the person what you are going to do.
    - Position the person comfortably.
  - Answer any questions they have.
  - Allow enough time for the person to feel comfortable with the decision they are making.
  This is an ethical approach to clinical decision making which recognises and respects patient autonomy and supports shared decision making. When patients are fully involved in decisions about their treatment, they are more likely to follow through with it. For example, shared decision making can help to avoid a situation which is quite common: that people fail to take prescribed medication, use it incorrectly, or simply stop using it.
6. **Support self-management** All people make decisions, take actions and manage factors that contribute to their health on a day-to-day basis. We can support them by sending reminders (i.e., by SMS) about future appointments, reminders to monitor their vision or eye health, and messages that prompt them to prevent eye problems and maintain their eye health. This can enable people to develop their knowledge to make informed decisions and effectively manage their own health.

In conclusion, people who are engaged in their own health care are more likely to:

- have a good experience of health care
- have information about their own health and have realistic expectations
- reach shared decisions about diagnoses and treatment that are right for them
- manage their care, adhere to treatment and have better health outcomes.
